# Significant plastic accumulation on the Cocos (Keeling) Islands, Australia

**DOI:** 10.1038/s41598-019-43375-4

**Published:** 2019-05-16

**Authors:** J. L. Lavers, L. Dicks, M. R. Dicks, A. Finger

**Affiliations:** 1 Institute for Marine and Antarctic Studies, University of Tasmania, 20 Castray Esplanade, Battery Point, TAS 7004 Australia; 2 Sea Shepherd Australia Marine Debris, PO Box 1215, Williamstown, VIC 3016 Australia; 3 Institute for Sustainable Industries & Liveable Cities, Victoria University, PO Box 14428, Melbourne, VIC 8001 Australia

**Keywords:** Conservation biology, Environmental impact

## Abstract

For over 60 years, our oceans have been a reservoir for exponentially increasing amounts of plastic waste. Plastic has been documented at all levels of the marine food web, from the deepest oceanic trenches to the most far-flung beaches. Here, we present data on the presence of significant quantities of plastic on the remote Cocos (Keeling) Island group, located 2,100 km off the northwest coast of Australia. From our comprehensive surveys of debris on the beach surface, buried, and beach-back vegetation, we estimate there are 414 million anthropogenic debris items, weighing 238 tonnes, currently deposited on the Cocos (Keeling) Island group. Of the identifiable items, ~25% were classified as disposable plastics, including straws, bags, and toothbrushes. Debris buried up to 10 cm below the surface is estimated to account for 93% (~383 million items) of all debris present on Cocos, the majority of which (~60%) is comprised of micro-debris (2–5 mm). In the absence of meaningful change, debris will accumulate rapidly on the world’s beaches. Small, buried items pose considerable challenges for wildlife, and volunteers charged with the task of cleaning-up, thus preventing new items from entering the ocean remains key to addressing this issue.

## Introduction

Global plastic production has increased exponentially over the last 60 years^1^. Nearly half of all plastic manufactured during this time (~8,300 million metric tons; Mt) was produced in the last 13 years (3,900 Mt) with ~40% of items entering the waste stream in the same year they were produced (e.g., single-use packaging)^2^. Insufficient or ineffective waste management contributed to an estimated 12.7 million Mt of plastic entering our oceans in 2010^2,3^ with a recent global estimate (5.25 trillion items)^4^ suggesting there are now more pieces of plastic in the ocean than there are stars in the Milky Way^5^. Numbers as large as these make it difficult to comprehend the scale of the issue and the feasibility of possible solutions^6,7^. Unfortunately, unless drastic steps are taken, the numbers and challenges will only grow, with the quantity of waste entering the ocean predicted to increase ten-fold by 2025^3^.

Once in the ocean, plastic items exposed to wave action and sunlight begin to fragment into small particles that persist for decades, perhaps centuries^8^. Therefore, the characteristics that make plastic such a popular material (e.g. durability, light-weight, low cost) also contribute to its abundance in the ocean, and to its role as a significant environmental threat^9^. Removing the significant quantities of plastic already in the ocean is not possible^10,11^, making the prevention of new items entering the ocean at their source critically important^12^.

Anthropogenic debris (hereafter ‘debris’) harms a diversity of aquatic wildlife directly via entanglement and ingestion^13^, as well as indirectly through exposure to plastic-associated chemicals and microbes^14–16^. There is an urgent need to quantify and mitigate these impacts, establish patterns of temporal change, and recognise plastic debris for what it is: a persistent, hazardous, and rapidly expanding environmental pollutant^17,18^.

Because there are few local sources of pollution and little human interference (i.e., no recreational beach users or debris removal via beach clean-ups), isolated islands with little or no human occupation can act as marine pollution monitors, providing unique insights into debris accumulation trends^19^. Here we present quantitative data from beach surveys of inhabited and uninhabited islands in the Cocos (Keeling) Island group (henceforth CKI). Using a comprehensive beach debris sampling method^19^, we highlight the ubiquitous and problematic nature of plastics on remote islands, especially that of “single-use” items, and the utility of beach survey data as a sentinel for the state of the world’s oceans.

## Methods

### Study site

**Figure 1:**
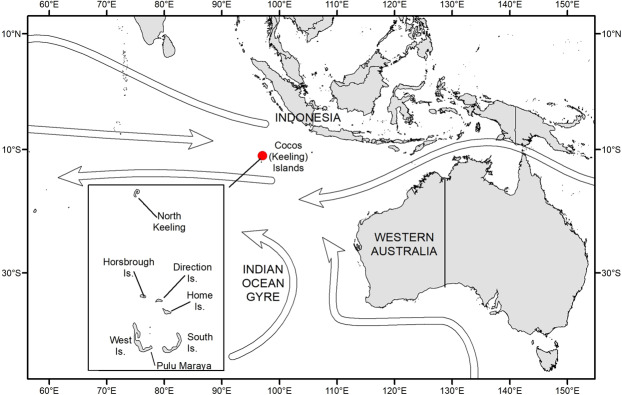
The location of the Cocos (Keeling) Islands. Arrows indicate the direction of major oceanic currents, including part of the Indian Ocean Gyre.

The Cocos (Keeling) Islands (CKI; 12°05′S, 96°53′E) comprise two small, mid-oceanic atolls (total land area 14 km^2^) located approximately 2,100 km north-west of Exmouth, Western Australia (Fig. 1) in the Indian Ocean. The islands are an Australian external territory. The much larger, southern atoll consists of a horse-shoe chain of 26 islands around a shallow, central lagoon with West and Home Island inhabited by ~600 people^20^. The northern atoll (North Keeling, designated as Pulu Keeling National Park) is a rarely visited, uninhabited island that is an important breeding site for seabirds^21^. Seven out of 27 islands in the CKI group were sampled during our 2017 survey. These seven islands account for 88% of the landmass of CKI (i.e., the remaining islands not surveyed were very small) and included both inhabited and uninhabited islands. Data were collected from two beaches (lagoon- and ocean-facing) per island to capture the variability in debris density, with additional beaches sampled on West and South Island (the largest islands in the CKI group). Where possible, transects or quadrats were also completed on a range of sediment types (e.g., sand, pebbles).

### Sample collection

**Figure 2:**
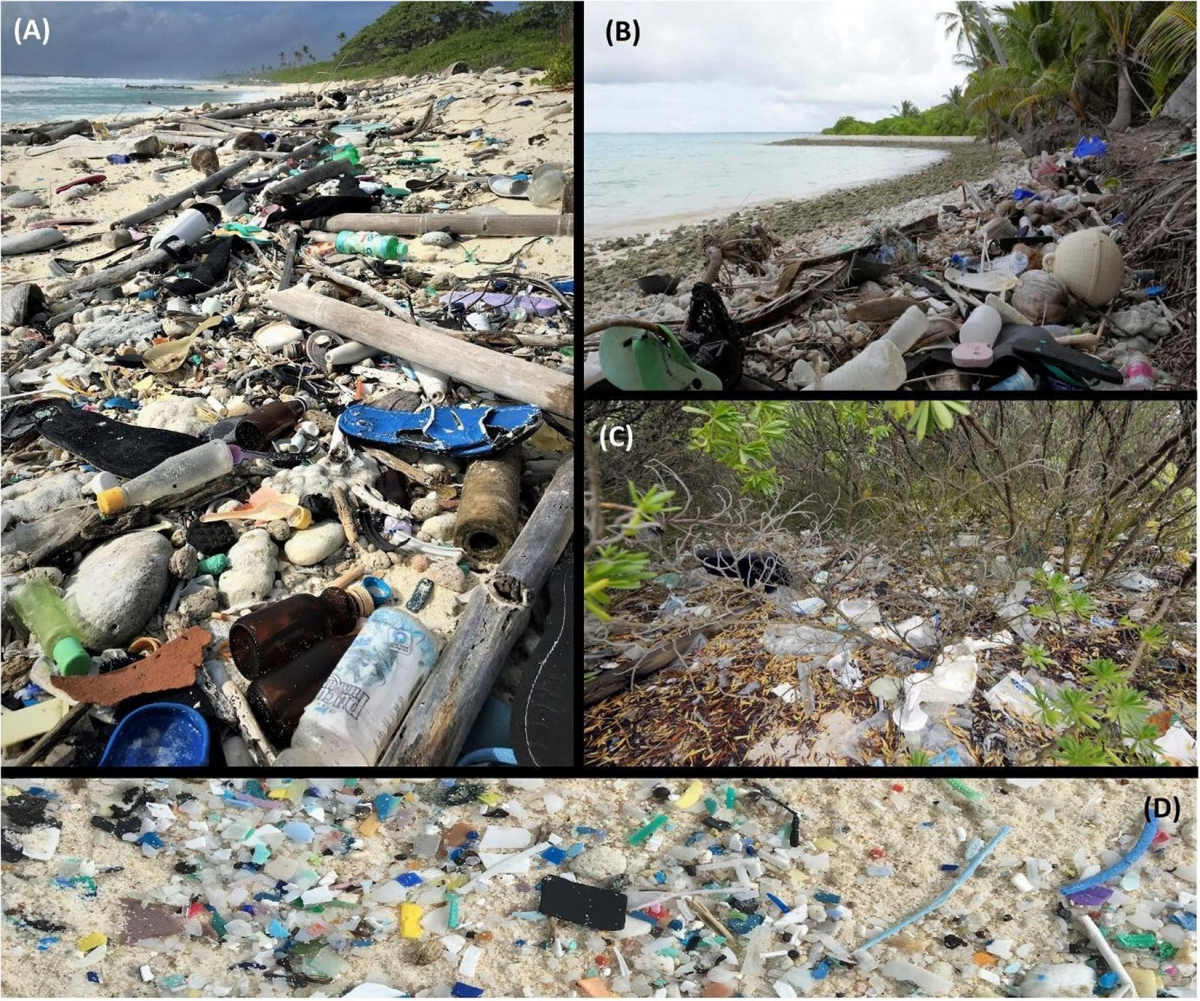
Anthropogenic debris on the Cocos (Keeling) Islands, March 2017. (**A**) eastern side of South Island, (**B**) north side of Direction Island, (**C**) beach-back vegetation along the north-east side of Home Island, (**D**) micro-plastics (primarily; 1–5 mm) along the eastern side of South Island.

The standing stock of accumulated micro- (2–5 mm) and macro-debris (>5 mm), including plastic, glass, wood, and metal items, was sampled along a total of 25 beaches (seven islands) of the CKI group (total sampled area 1,110 m^2^). Most sites were surveyed from 13–24 March 2017, however, due to the difficulty in accessing North Keeling, it was not possible to survey this island until 21 September 2017. To ensure beach debris data collected on CKI were comparable with the data from other remote islands, the sampling methods used on CKI were identical to those developed for Henderson Island in the South Pacific^19^. As shown in Fig. 2 of Lavers and Bond^19^, we used three different transect and quadrat designs aimed at providing specific types of data: (1) we sampled surface beach debris along fifteen 30 × 6 m transects which covered most of the distance from the water’s edge to the start of the vegetation. Within this area, the transect was positioned with the middle 2 m wide strip centred over the high tide mark (i.e., area of known debris accumulation). (2) We collected data on the density of debris located within the low-lying beach-back vegetation using twelve 10 × 2 m transects on five islands. The transects were run perpendicular to the water’s edge, extending 10 m from the vegetation line toward the centre of each island. Only macro-debris (≥5 mm) was recorded in the beach-back area because of the difficulty in detecting smaller items amongst vegetation. (3) Buried debris (to a depth of 10 cm) was sieved from all sediment excavated in seven paired quadrats following the method of Kusui and Noda^22^, with each pair comprising one quadrat along the high tide line, and one located approximately 2 m below the vegetation line. At each quadrat, a 0.4 × 0.4 m wooden frame was inserted into the sand, and the contents of the frame were exhumed to a depth of 10 cm. Items located above a depth of 1 cm were considered to be surface debris and were excluded. The inner island, beyond the beach-back transect area, was not surveyed and has not been included in any extrapolations.

All debris items encountered in transects or quadrats were counted, sorted by type, and weighed using an electronic balance to 0.001 g (for micro-debris 2–5 mm) or 1 g using a spring balance (for macro-debris ≥5 mm). We used the same standard debris type categories as Lavers and Bond^20^, including glass, foam, metal, hard plastic fragments, film (e.g., soft plastics such as bags), and threads (e.g., rope and fishing line). Additional sub-categories included readily identifiable items or those commonly reported in the recent literature (e.g., industrial resin pellet (“nurdle”), melted plastic, shoes, cigarette lighters, and toothbrushes). All values are presented as mean ± SD unless specified otherwise.

### Statistical analysis

The total amount of debris present on each island in the Cocos (Keeling) Island group in 2017 was estimated in five steps. (1) The total beach area and perimeter for each island was measured using aerial imagery in Google Earth Pro. (2) We then multiplied the mean density of items recorded from beach transects by the total beach area to estimate the total debris visible on the beach surface of each island. (3) Next, we estimated the total amount of debris buried to a depth of 10 cm on each island based on the mean buried density recorded from quadrats and total beach surface area of each island. (4) The quantity of debris present within a 10 m wide zone of beach-back vegetation was estimated using the mean density values generated from 10 m transects running perpendicular from the beach edge multiplied by the perimeter of each island. (5) Missing data on the density and/or mass of debris (e.g., North Keeling) were estimated based on the combined mean of the remaining islands in the CKI group. Finally, the total number and mass of items recorded on the seven islands surveyed (representing 88% of the landmass) were extrapolated using mean debris numbers and mass to provide estimates for the entire CKI group.

## Results

### Overall

We recorded a total of 23,227 anthropogenic debris items, weighing a total of 96.67 kg. Based on these data, and the density estimates detailed below, we estimate there were 414 million pieces of anthropogenic debris weighing 238 tonnes present on the entire Cocos (Keeling) Islands group in 2017 (i.e., extrapolated to include all 27 islands, Table 1).

**Table 1:** Estimates of the number (n) and mass of debris items (kg) present on the Cocos (Keeling) Island group in 2017.

Island	Beach surface area (m^2^)	Island perimeter (km)	Estimated number of items (n)			Estimated mass of items (kg)			Estimated island total	
			Beach surface	Beach-back	Buried (1–10 cm)	Beach surface	Beach-back	Buried (1–10 cm)	Mass (kg)	Number (n)
Direction Is.	17,033	3,237	129,621	969,482	12,109,398^d^	4,655	6,312	1,924^g^	12,891	13,208,501
Home Is.	27,498	6,285	913,850	3,221,063	8,850,919	1,116	26,287	365	27,768	12,985,832
Horsburgh Is.	21,059	4,159	548,798	486,603	14,971,633^d^	1,923	6,758	2,378^g^	11,059	16,007,034
North Keeling	85,035	5,737	1,708,873^a^	1,159,046^c^	60,454,570^d^	9,932^e^	10,491^f^	9,603^g^	30,026	63,322,489
Pulu Marya Is.	3,522	593	79,827^b^	13,491	2,503,922^d^	411^e^	83	398^g^	892	2,597,240
South Is.	105,820	23,835	2,154,025	4,815,385^c^	157,903,281	16,455	43,586^f^	30,754	90,795	164,872,691
West Is.	255,880	25,710	7,302,176	1,501,464	81,561,750	5,970	32,048	8,929	46,947	90,365,390
**TOTAL** (**surveyed islands**)	**515**,**847**	**69**,**556**	**12**,**837**,**170**	**12**,**166**,**534**	**338**,**355**,**473**	**40**,**462**	**125**,**565**	**54**,**351**	**220**,**378**	**363**,**359**,**177**
Islands not sampled (n = 20)	64,201	15,629	1,455,133^c^	3,157,527^c^	45,642,898^d^	7,499^e^	2,858^f^	7,250^g^	17,607	50,255,558
**TOTAL** (**entire CKI group**)	**580**,**048**	**85**,**185**	**14**,**292**,**303**	**15**,**324**,**061**	**383**,**998**,**371**	**47**,**961**	**128**,**423**	**61**,**601**	**237**,**985**	**413**,**614**,**735**

^a^Mean density for micro-debris (12.13 items m^−2^) was applied.

^b^Mean density for visible debris on the beach surface (22.67 items m^−2^) was applied.

^c^Mean density for macro-debris in the beach-back (20.20 items m^−2^) was applied.

^d^Mean density for buried debris (710.94 items m^−2^) was applied.

^e^Mean mass for beach surface debris (116.80 g m^−2^) was applied.

^f^Mean mass for beach-back debris (182.87 g m^−2^) was applied.

^g^Mean mass for buried debris (112.93 g m^−2^) was applied.

### Surface debris

The mass and number of debris items recorded across all 15 beach surface transects (868 m^2^) was 70.73 kg (excluding North Keeling samples, which were not weighed) and 18,928 items, respectively. Mean density of visible debris on the beach surface ranged from 4.72 items m^−2^ (Gun Beach 2, West Island) to 55.67 items m^−2^ (North Cove, Home Island; Table 2; overall mean 21.68 ± 19.01 items m^−2^). About half of the collected surface debris by number was comprised of microplastics (51.35 ± 9.31%; Fig. 2, panel D). Extrapolated to the total beach area for all of CKI, an estimated 14.29 million pieces of visible debris weighing 47.96 tonnes were present on beach surfaces of the CKI group (Table 1).

**Table 2:** Mean density (items m^−2^ ± SD) and mass (g m^−2^) of debris items recorded in transects and quadrats on Cocos (Keeling) Island group.

Island/Site	Density (items m^−2^)					Mass (g m^−2^)				
	Surface			Buried (1–10 cm)	Beach-back	Surface			Buried (1–10 cm)	Beach-back
	-macro	-micro	-all			-macro	-micro	-all		
**Direction Island**	**5**.**69** ± **0**.**05**	**3**.**85**	**7**.**61** ± **2**.**67**^**c**^	**N/A**	**29**.**95**	**273**.**24** ± **377**.**26**	**0**.**12**	**273**.**30** ± **377**.**18**	**N/A**	**195**.**00**
NE tip	5.73	N/A^a^	5.73^c^	N/A	N/A	540.00	N/A^1^	540.00^2^	N/A	N/A
Northside	5.65	3.85	9.50	N/A	N/A	6.48	0.12	6.59	N/A	N/A
NW cove	N/A	N/A	N/A	N/A	29.95	N/A	N/A	N/A	N/A	195
**Home Island**	**16**.**03** ± **13**.**65**	**17**.**20** ± **18**.**08**	**33**.**23** ± **31**.**73**	**321**.**88** ± **415**.**43**	**51**.**25**	**40**.**15** ± **48**.**91**	**0**.**44** ± **0**.**54**	**40**.**58** ± **49**.**45**	**13**.**28** ± **5**.**52**	**418**.**25**
North Cove	25.68	29.98	55.67	615.63	51.25	74.73	0.82	75.55	17.19	418.25
South Beach	6.38	4.42	10.80	28.13	N/A	5.56	0.06	5.62	9.38	N/A
**Horsburgh Is**.	**13**.**37** ± **12**.**24**	**12**.**69** ± **6**.**28**	**26**.**06** ± **18**.**51**	**N/A**	**11**.**7**	**91**.**20** ± **47**.**95**	**0**.**10** ± **0**.**14**	**91**.**30** ± **48**.**09**	**N/A**	**162**.**50**
North east	22.02	17.13	39.15	N/A	11.7	57.29	0.00	57.29	N/A	162.50
South west	4.72	8.25	12.97	N/A	N/A	125.10	0.20	125.3	N/A	N/A
**North Keeling**	**8**.**07** ± **4**.**27**	**N/A**	**8**.**07** ± **4**.**27**^**c**^	**N/A**	**N/A**	**N/A**	**N/A**	**N/A**	**N/A**	**N/A**
Lagoon entry	5.05	N/A^b^	5.05^c^	N/A	N/A	N/A	N/A	N/A	N/A	N/A
Leeward	11.08	N/A^b^	11.08^c^	N/A	N/A	N/A	N/A	N/A	N/A	N/A
**Pulu Marya Is**.	**N/A**	**N/A**	**N/A**	**N/A**	**2**.**28** ± **3**.**86**	**N/A**	**N/A**	**N/A**	**N/A**	**13**.**94** ± **6**.**04**
Lagoon	N/A	N/A	N/A	N/A	0.70	N/A	N/A	N/A	N/A	7.75
Sandy Spit	N/A	N/A	N/A	N/A	8.05	N/A	N/A	N/A	N/A	20.50
Landing	N/A	N/A	N/A	N/A	0.05	N/A	N/A	N/A	N/A	10.00
Pondok	N/A	N/A	N/A	N/A	0.30	N/A	N/A	N/A	N/A	17.50
**South Island**	**12**.**23** ± **13**.**35**	**8**.**13** ± **6**.**09**	**20**.**36** ± **9**.**60**	**1492**.**19** ± **1434**.**10**	**N/A**	**155**.**33** ± **127**.**72**	**0**.**18** ± **0**.**11**	**155**.**50** ± **127**.**78**	**290**.**62** ± **150**.**26**	**N/A**
Central SE	23.35	4.23	27.58	2506.25	N/A	287.42	0.28	287.70	184.38	N/A
South west	4.47	5.00	9.47	N/A	N/A	32.48	0.18	32.66	N/A	N/A
South east	8.87	15.15	24.02	478.13	N/A	146.08	0.07	146.15	396.88	N/A
**West Island**	**9**.**76** ± **8**.**17**	**18**.**78** ± **19**.**68**	**28**.**54** ± **27**.**29**	**318**.**75** ± **417**.**72**	**5**.**84** ± **5**.**99**	**22**.**79** ± **16**.**19**	**0**.**54** ± **0**.**59**	**23**.**33** ± **16**.**60**	**34**.**90** ± **18**.**51**	**124**.**65** ± **76**.**25**
Gun Beach 1	3.60	1.50	5.10	50.00	N/A	18.16	0.02	18.18	23.44	N/A
Gun Beach 2	2.37	2.35	4.72	106.25	N/A	11.52	0.07	11.58	25.00	N/A
Jetty Beach T16	N/A	N/A	N/A	N/A	8.85	N/A	N/A	N/A	N/A	182.25
Jetty Beach T17	N/A	N/A	N/A	N/A	2.45	N/A	N/A	N/A	N/A	55.75
Jetty Beach T18	N/A	N/A	N/A	N/A	0.90	N/A	N/A	N/A	N/A	28.00
Jetty Beach T19	N/A	N/A	N/A	N/A	2.00	N/A	N/A	N/A	N/A	182.25
Scout Park 1	13.75	39.20	52.95	800.00	N/A	14.75	0.92	15.67	56.25	N/A
Scout Park 2	19.33	32.05	51.38	N/A	N/A	46.73	1.17	47.90	N/A	N/A
Monument	N/A	N/A	N/A	N/A	15.00	N/A	N/A	N/A	N/A	175.00

^a^NE tip is a cobble beach, surface sampling of micro-debris was not possible.

^b^No micro-debris sampling undertaken at North Keeling due to logistical problems.

^c^This is an underestimation, as it only includes macro-debris. Micro-debris sampling was not undertaken at this site.

### Buried debris

Total buried debris (top 10 cm) recorded in 14 quadrats from seven sites on three islands (2.24 m^2^) accounted for only 6.32% of all beach debris items collected by number (n = 1,467) and 0.32% by weight (0.23 kg). Mean density of buried items (both micro- and macro-debris) varied from 28.13 items m^−2^ (South Beach, Home Island) to 2,506.25 items m^−2^ (Central SE, South Island; overall mean 654.91 ± 869.97 items m^−2^), while the mean mass of items ranged from 0.01 kg m^−2^ (Home Island) to 0.29 kg m^−2^ (South Island; Table 2). Between 10–70× more debris items m^−2^ were found buried compared to items visible on the surface of beaches, and 76.42 ± 10.99% of buried debris items were comprised of micro-debris. The estimated total number of buried debris items for CKI is 383.99 million pieces weighing 61.60 tonnes (Table 1).

### Beach-back debris

The 12 beach-back transects completed on five islands (240 m^2^) recovered 2,622 items, weighing 25.71 kg. The mean density of beach-back debris (macro only) ranged from 2.28 items m^−2^ (Pulu Marya Island) to 51.25 items m^−2^ on (Home Island; Table 2 and Fig. 2, panel C; overall mean 10.93 ± 15.39 items m^−2^). Data from Home and Horsburgh Islands indicated the average mass of macro-debris found in beach-back areas was 3–6× heavier than on the beach surface. Extrapolated to the total beach-back area of the island group, we estimate that there were 15.32 million debris items present in the beach-back, weighing a total of 128.42 tonnes, across the CKI (Table 1).

### Debris composition

Plastic items accounted for 95.41% (n = 22,161) of all debris recorded, followed by foam (3.96%, n = 919). All other debris types combined (glass, metal, wood and fabric) made up 0.63% (n = 147) of the total count. Plastic fragments were the most common item encountered on CKI beaches (69.34%, n = 16,106). Of the identifiable items recorded during the survey, 9.84% were categorised as disposable single-use items (n = 2,285), 3.84% as resin pellets (‘nurdles’, n = 891) and 1.57% as fishing-related (n = 365). The most common identifiable consumer items were bottle caps and lids (n = 840), shoes (predominantly ‘flip-flops’, n = 549) and plastic drinking straws (n = 235).

## Discussion

Results of the first, comprehensive survey of debris on the Cocos (Keeling) Islands in 2017 indicated there are an estimated 413.6 million pieces of debris weighing 238 tonnes (Table 1) distributed across these remote, tropical islands. While these numbers are among the highest reported on remote islands^19,23^ the data underestimate the true amount of debris present on CKI as we were unable to sample all possible debris sources. For example, we excluded items buried >10 cm below the surface and were unable to survey some debris ‘hotspots’ within the atoll, including the south-east side of South Island (Fig. 2; panel A and D) due to accessibility issues (i.e., unfavourable tides, vessel mechanical failure). As a result, our data on debris densities for CKI are conservative and values should be interpreted as minimum estimates.

The density of debris appeared to vary according to a range of factors on CKI, depending on the island. For example, the density of macro-debris on Horsburgh Island ranged from 4.72 to 22.02 items m^−2^ on the lagoon-facing and ocean-facing beaches, respectively (Table 2). However, as Horsburgh Island is located at the northern end of the atoll (Fig. 1), it is partially sheltered from ocean currents. In contrast, the ocean-facing beaches on South Island face directly into the predominant currents in this region (Fig. 1), which tend to flow in a westerly direction^24^ and cause vast quantities of debris to accumulate on these exposed beaches (Fig. 2, panel A & D).

Most studies of beach debris focus on large, visible plastics because these items are readily observed, easily collected, and can provide additional information when intact (e.g., country of manufacture)^25^. In contrast, paired data on micro- and macro-debris are limited, primarily due to the difficulty in identifying small items^25–27^. On CKI, where both micro- and macro-debris were recorded, micro-debris accounted for 60.29 ± 16.72% of items, comparable to Henderson Island in the remote South Pacific (~62% of beach-washed items were micro-debris)^19^. The removal of micro-debris from beaches poses a significant challenge even at small scales, due to the time required to separate plastics <5 mm from sediment and other organic materials^25^. As a result, large-scale estimates of debris accumulation generated by researchers and citizen scientists (e.g., International Coastal Cleanup, Clean-up Australia Day) rarely account for micro-debris items, meaning the data commonly referenced by media and policy makers are very conservative.

Based on the estimated totals for the CKI group (Table 1), the quantity of debris predicted to be buried 1–10 cm below the beach surface (n = 338,355,473 items) is 26x greater than the amount of debris visible on the beach surface (3.80%, n = 12,868,379 items). Again, this suggests global debris surveys, the majority of which are focused solely on surface debris, have drastically underestimated the scale of debris accumulation. These findings highlight a growing need for the development of effective policy and mitigation, which are currently focused primarily on localised clean-up of visible debris, and raises questions regarding the potential impact of buried plastic on wildlife nesting or living within beach sediments, such as sea turtles, crustaceans, and meiofauna. Furthermore, the removal of buried debris would require major mechanical disturbance of sediments, with potentially significant environmental impacts on inhabiting biota. Cozar *et al*.^28^ suggested the deposition of millimetre-sized plastic on shorelines was unlikely to explain the gap in size distribution or global surface load of floating plastic debris. However, these conclusions were largely based on studies of surface macro-debris. On CKI, 78.12% of the estimated 384 million buried items (Table 1) were 2–5 mm in size, thus new, more comprehensive data on the deposition of debris, including micro- and buried items on remote islands, may be of increasing importance when interpreting patterns observed at-sea.

While some plastic types (e.g., fragments) are ubiquitous and reported in most beach surveys, other items appear to accumulate only in specific areas. For example, fishing activities are often implicated with respect to marine debris, with around 15–60% of items recorded at sea and washed up on beaches attributed to commercial or recreational fishing^29^. In contrast, fishing-related debris was relatively uncommon on CKI (1.6% of items recorded, n = 365; Table 3), compared to other remote islands where 8–46% of beach-washed items were fishing related^19,23^. Instead, shoes (n = 549) and “single-use” or “disposable” consumer items (e.g., food packaging, drink bottles, straws, plastic cutlery, bags, toothbrushes; n = 2,285; Fig. 2, Table 3) accounted for nearly 25% of the debris present on CKI beaches. Thus, the debris on CKI seems to mirror global data on plastic production^2^, and highlights a worrying trend in the production and discharge of single-use products^2,30^.

**Table 3:** Number (*n*) and frequency of occurrence (FO) of major categories of anthropogenic debris on the Cocos (Keeling) Islands in 2017 (area surveyed 1110 m^2^, total items recorded 23,227) relative to uninhabited Henderson Island (Pitcairn Islands Group, 2015, area surveyed 1310 m^2^, total items recorded 53,164).

Item	Cocos (Keeling) Islands		Henderson Island	
	*n*	FO	*n*	FO
*Single-use items*				
Food packaging	1,158	0.050	1	<0.001
Caps and lids	840	0.036	486	0.008
Plastic bottle	145	0.006	115	0.002
Plastic bag (and bits)	367	0.016	60	0.001
Drinking straw	235	0.010	10	<0.001
Cigarette lighter	86	0.004	3	<0.001
Cotton bud stick	206	0.089	—	—
Toothbrush	21	0.009	2	<0.001
*Fishing related*				
Rope	297	0.013	3,336	0.054
Plastic strapping	17	<0.001	642	0.010
Crates (and bits)	7	<0.001	245	0.004
Fishing line	4	<0.001	220	0.004
*Other*				
Plastic fragment	16,106	0.693	48,121	0.791
Resin pellet (‘nurdle’)	891	0.038	6,774	0.111
Shoes	549	0.024	4	<0.001

Our excessive and unrelenting demand for plastics, coupled with ineffective policy and waste management, has resulted in myriad negative effects on marine, freshwater, and terrestrial environments, including entanglement and ingestion of debris, and subsequent exposure to plastic-associated chemicals^13,31–33^. Damaged environmental aesthetics, with consequent impacts on tourism, are also widely documented^34,35^. CKI is touted as “Australia’s last unspoilt paradise”, with tourism a primary source of income for the local community^36^. However, the impact of debris on tourism and CKI’s beaches, is increasingly difficult to avoid. For example, Cossie’s Beach on the south side of Direction Island was recently named “Australia’s top stretch of sand”^37^, yet beaches <200 m away on the northern side of the island exhibit some of the highest debris densities in the CKI group (Table 1, Fig. 2 panel B). Correspondingly, pollution of the marine environment has been identified as an emerging management issue for CKI (action 3.2.2)^38^.

**Figure 3:**
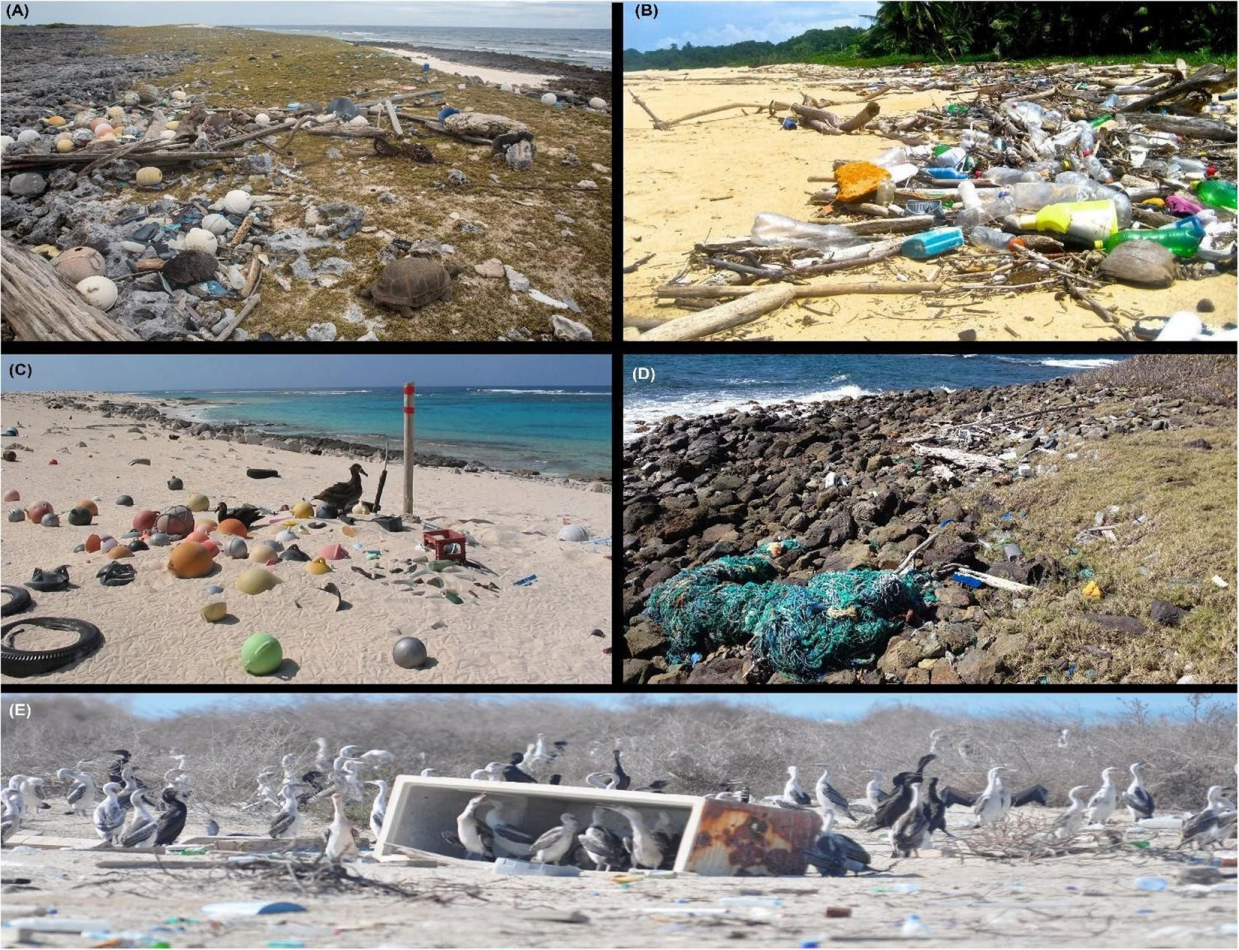
Anthropogenic debris is widespread on remote, uninhabited islands. (**A**) Aldabra Atoll, north-west Indian Ocean, 2017. (**B**) Holandes Cays, San Blas Islands, Panama, 2013. (**C**) Laysan Island, North Pacific Ocean, 2004. (**D**) Catholic Island, Caribbean Sea, 2014. (**E**) Siniya Island, United Arab Emirates, 2013.

Sadly, the situation on the Cocos (Keeling) Islands is not unique, with significant quantities of debris documented on islands and coastal areas from the Arctic to the Antarctic (see Fig. 3)^39,40^. Together, these islands and coastal areas reflect the acute symptoms of an otherwise rapidly increasing environmental hazard. Legal protection (e.g., World Heritage listing, marine/national park designation) and a lack of human activity has not afforded remote sites like CKI and Henderson Island protection from debris washing up on their shores^19^. On CKI, infrastructure (e.g., waste management) has also failed to protect these islands against debris accumulation. So, what will provide effective protection? With the quantity of debris entering the world’s oceans predicted to increase by an order of magnitude by 2025^3^, that question is now urgent. Clean-up events focused solely on surface debris of a single remote island can take months to negotiate, require significant staff time, cost tens of thousands of dollars, and typically deposit the collected debris into landfill (authors’ personal observations). With an estimated 2,000 oceanic islands worldwide, and thousands of new plastic items washing up on remote beaches every day^19^, clean-ups cannot keep pace. The beaches of CKI are already home to an estimated 373,000 toothbrushes and 977,000 shoes - equivalent to what the CKI community would produce as waste in ~4000 years. In the absence of rapid and meaningful change, anthropogenic debris will accumulate on beaches^19^, with impacts increasingly felt by biodiversity^41,42^ and marine plastic mitigation will remain a perpetual game of catch-up. Mitigation initiatives, including policy, should be mindful of the challenges faced by remote islands, and the communities that reside there. For example, CKI has struggled to identify an appropriate location for landfill (a challenge on many, low-lying islands) and is unable to export recyclable items to the Australian mainland due to complex biosecurity legislation^43^. Prevention is key, and for that, a multi-prong approach is urgently required, including significant investment in strategies designed to limit plastic production and consumption (e.g., widespread bans on single-use items), and effective waste management that prevents entry of plastic items into the ocean at the source (‘source reduction’)^44^.
