# Author Correction: Significant plastic accumulation on the Cocos (Keeling) Islands, Australia

**DOI:** 10.1038/s41598-023-34610-0

**Published:** 2023-05-12

**Authors:** J. L. Lavers, L. Dicks, M. R. Dicks, A. Finger

**Affiliations:** 1 Institute for Marine and Antarctic Studies, University of Tasmania, 20 Castray Esplanade, Battery Point, TAS 7004 Australia; 2 Sea Shepherd Australia Marine Debris, PO Box 1215, Williamstown, VIC 3016 Australia; 3 Institute for Sustainable Industries & Liveable Cities, Victoria University, PO Box 14428, Melbourne, VIC 8001 Australia

Correction to: *Scientific Reports* https://doi.org/10.1038/s41598-019-43375-4, published online 16 May 2019

This Article contains errors.

In Figure 1 the Leeuwin current is incorrectly shown as flowing south to north, instead of north to south. The correct Figure 1 and accompanying legend appear below.

**Figure 1:**
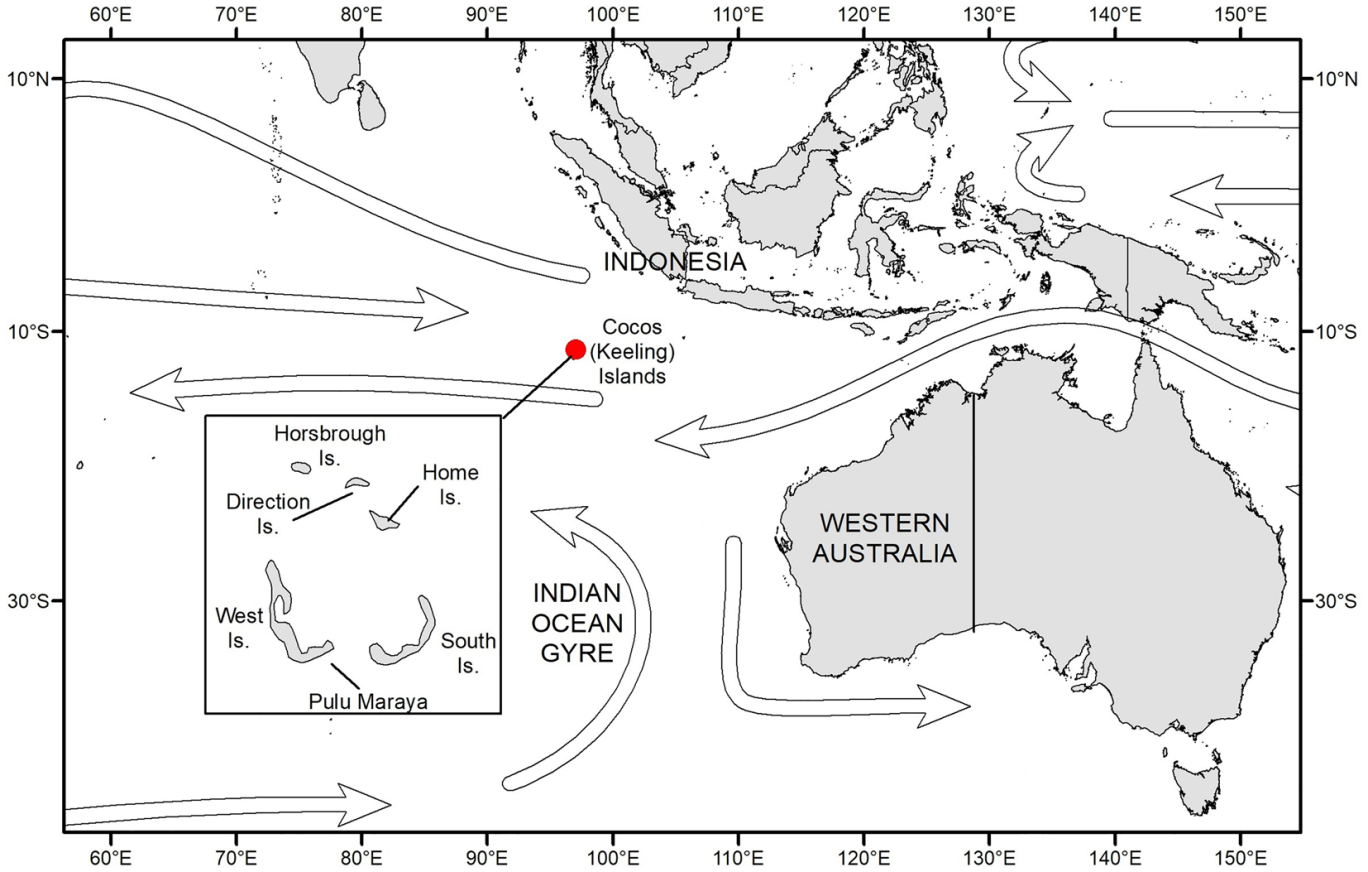
The location of the Cocos (Keeling) Islands. Arrows indicate the direction of major oceanic currents, including part of the Indian Ocean Gyre.

In Table 1, the measurement for Island perimeter is incorrectly recorded as 'km'. The correct unit is metres (m).

**Table 1:** Estimates of the number (n) and mass of debris items (kg) present on the Cocos (Keeling) Island group in 2017.

			**Estimated number of items (n)**			**Estimated mass of items (kg)**			**Estimated island total**	
**Island**	**Beach surface area (m**^**2**^**)**	**Island perimeter (m)**	**Beach** **surface**	**Beach-back**	**Buried** **(1 to 10 cm)**	**Beach surface**	**Beach-back**	**Buried (1–10 cm)**	**Mass (kg)**	**Number (n)**
Direction Is	17,033	3,237	129,621	969,482	12,109,398^d^	4,655	6,312	1,924^ g^	12,891	13,208,501
Home Is	27,498	6,285	913,850	3,221,063	8,850,919	1,116	26,287	365	27,768	12,985,832
Horsburgh Is	21,059	4,159	548,798	486,603	14,971,633^d^	1,923	6,758	2,378^ g^	11,059	16,007,034
North Keeling	85,035	5,737	1,708,873^a^	1,159,046^c^	60,454,570^d^	9,932^e^	10,491^f^	9,603^ g^	30,026	63,322,489
Pulu Marya Is	3,522	593	79,827^b^	13,491	2,503,922^d^	411^e^	83	398^ g^	892	2,597,240
South Is	105,820	23,835	2,154,025	4,815,385^c^	157,903,281	16,455	43,586^f^	30,754	90,795	164,872,691
West Is	255,880	25,710	7,302,176	1,501,464	81,561,750	5,970	32,048	8,929	46,947	90,365,390
**TOTAL (surveyed islands)**	**515,847**	**69,556**	**12,837,170**	**12,166,533**	**338,355,473**	**40,462**	**125,565**	**54,351**	**220,378**	**363,359,177**
Islands not sampled (n = 20)	64,201	15,629	1,455,133^c^	3,157,527^c^	45,642,898^d^	7,499^e^	2,858^f^	7,250^ g^	17,607	50,255,558
**TOTAL (entire CKI group)**	**580,048**	**85,185**	**14,292,302**	**15,324,061**	**383,998,371**	**47,961**	**128,423**	**61,601**	**237,985**	**413,614,735**

^a^Mean density for micro-debris (12.13 items m^−2^) was applied. ^b^Mean density for visible debris on the beach surface (22.67 items m^−2^) was applied. ^c^Mean density for macro-debris in the beach-back (20.20 items m^−2^) was applied. ^d^Mean density for buried debris (710.94 items m^−2^) was applied. ^e^Mean mass for beach surface debris (116.80 g m^−2^) was applied. ^f^Mean mass for beach-back debris (182.87 g m^−2^) was applied. ^g^Mean mass for buried debris (112.93 g m^−2^) was applied.

